# Vault RNAs (vtRNAs): Rediscovered non-coding RNAs with diverse physiological and pathological activities

**DOI:** 10.1016/j.gendis.2023.01.014

**Published:** 2023-03-23

**Authors:** Mahsa Aghajani Mir

**Affiliations:** Deputy of Research and Technology, Health Research Institute, Babol University of Medical Sciences, Babol 47176-4774, Iran

**Keywords:** ncRNA, Vault complex, Vault RNA, vtRNA, viral infection, tumorigenesis

## Abstract

The physicochemical characteristics of RNA admit non-coding RNAs to perform a different range of biological acts through various mechanisms and are involved in regulating a diversity of fundamental processes. Notably, some reports of pathological conditions have proved abnormal expression of many non-coding RNAs guides the ailment. Vault RNAs are a class of non-coding RNAs containing stem regions or loops with well-conserved sequence patterns that play a fundamental role in the function of vault particles through RNA-ligand, RNA–RNA, or RNA-protein interactions. Taken together, vault RNAs have been proposed to be involved in a variety of functions such as cell proliferation, nucleocytoplasmic transport, intracellular detoxification processes, multidrug resistance, apoptosis, and autophagy, and serve as microRNA precursors and signaling pathways. Despite decades of investigations devoted, the biological function of the vault particle or the vault RNAs is not yet completely cleared. In this review, the current scientific assertions of the vital vault RNAs functions were discussed.

## Introduction

Recently, the importance of diverse and surprising types of non-protein coding RNA (ncRNA) molecules have been widely recognized. In addition, all transcripts analyzed to date consist of two different classes of RNA molecules, mRNAs, which are generally translated into proteins by a series of ribosomes, and ncRNAs, which carry out cellular functions at the RNA level.^1^ In general, the existence of ncRNAs has been observed in all three kingdoms (Archaea, Bacteria, and Eukarya) and their number increases as organisms become more complex.^1^^,^^2^ Deep sequencing techniques have enabled a complete understanding of cellular transcriptomes, and it has been shown that primitive single-celled eukaryotes (*e.g.*, yeast) or prokaryotes contain fewer ncRNA genes than multicellular eukaryotes. Additionally, it has been estimated mammals encode around 100,000 ncRNAs. However, this knowledge of ncRNA is essential for understanding the complexity of mammals compared to “lower organisms” despite their slightly greater number of protein-coding genes.^2^, ^3^, ^4^, ^5^, ^6^

**Table 1:** A summary of validated dysregulated vault RNAs in different cellular processes.

ncRNA	Patients and cells tested	Related process	Description	Reference
vtRNA1-1	Animal models (mouse, rabbit, guinea pig) and cortical neuron culture	Synapse formation	hvtRNA1-1 increases the expression of synaptic marker proteins, ERKs phosphorylation, and the number of PSD-95 and synapsin I, thus modifying the modulation of the MAPK signaling pathway can improve synapse formation.	^95^,^96^
	Huh-7 cell line, murine cells	Autophagy	vtRNA1-1 contributes to the regulation of the autophagic flux, by directly interacting with the autophagy receptor protein SQSTM1/p62.	^68^,^137^
	EBV-infected human B cells	Viral infection	Ectopic expression of vtRNA1-1 leads to increased viral establishment and reduced apoptosis in B cells, leaving cells vulnerable to efficient EBV infection.	^66^
	HeLa cell line	Apoptotic signaling	The PI3K/Akt pathway and the ERK1/2-MAPK cascade are dysregulated in the vtRNA-1 knockout cell lines, thus eliciting increased levels of apoptosis in prolonged starvation.	^70^
	SCs skin	Cellular differentiation	Methylated vtRNA1-1 prevents the binding of SRSF2 and NSUN2 to its putative binding site spanning methylated C69, which in turn inhibits epidermal differentiation.	^155^,^158^
	HCC	Multidrug resistance	vtRNA1-1 knockdown in HCC cells leads to lysosomal compartment dysfunction by inhibiting TFEB nuclear translocation and increasing activation of the MAPK, resulting in lysosome-mediated resistance to chemotherapy.	^137^
	BCC line	Multidrug resistance and cell proliferation	vtRNA1-1 releases the PSF protein, allowing the GAGE6 oncogene to become activated and thereby inducing drug resistance by promoting cell proliferation.	^56^
vtRNA1-3	MDS	Cell proliferation	vtRNA1-3 promoter hypermethylation is associated with a decreased overall survival of MDS.	^87^
vtRNA2-1 (nc886)	AML	Cell proliferation	vtRNA2-1 promoter hypermethylation in AML induces pPKR down-regulation, which strikingly has a significantly poorer outcome in AML patients.	^86^
	GC	Cell proliferation	nc886 inhibits cell proliferation in GC cells via suppression of oncogenes such as FOS, NF-κB, and MYC.	^55^
	PCa	Cell proliferation	Hypermethylation of nc886 promoter in tumor tissue correlates with clinical staging of PCa, including biochemical recurrence, clinical T-value, and Gleason scores through increased cell proliferation and invasion.	^44^,^179^
	HCC	Cell proliferation	Hypermethylation of the vtRNA2-1 promoter is increased in stage III HCC tumors compared with stage I & II tumors and is also significantly associated with HCC tumor recurrence.	^90^
	PD	Cell proliferation	Hypermethylation of 15 CpG sites in the vtRNA2-1 gene can be associated with PD.	^91^
	T2DM	Glycemic response	Hypomethylation of vtRNA2-1 becomes considerably related to a poor glycemic reaction to GLP-1 treatment for T2DM.	^98^
	IgA nephropathy	Cell proliferation	Hypermethylation of vtRNA2-1 in IgAN patients led to a decreased CD4^+^ T-cell proliferation following TCR stimulation and to the overexpression of TGFβ.	^100^
	HaCaT cells	Sensitivity to UVB	nc886 expression decreases by UVB radiation, while PKR phosphorylation thru MAPKs will increase, and will increase the expression of inflammatory cytokines, MMP-9, and COX-2, which in the end boost inflammatory responses.	^105^
	SLE	Cell proliferation	Overexpression of vtRNA2-1 on B cells is associated with SLE through specific binding to PKR.	^180^
	Pregnant women	Preterm birth	Hypermethylation of the vtRNA2-1 promoter results in an increased risk of PTB caused by the pro-inflammatory cytokines.	^181^
	RCC	Apoptotic signaling	Overexpression of nc886 promotes A-498 cell proliferation and invasion and inhibits cell apoptosis by activating JAK2/STAT3 signaling.	^151^
	CCC	Multidrug resistance	E2F1 promotes nc886 transcription and turns MVP expression sufficiently to drive drug resistance in cervical cancer cells.	^174^
svtRNA2-1a	PD	Cell proliferation	Up-regulation of svtRNA2-1a in PD contributes to the disruption of gene expression networks, underlying metabolic impairments, and cellular dysfunction.	^94^
vtRNA2-1-5p	CSCC	Apoptotic signaling	Overexpression of vtRNA2-1-5p in human CSCC increases cervical cancer cell invasion, proliferation, and tumorigenicity while decreasing apoptosis and p53 expression.	^150^
svRNAb	HepG2 cells	Multidrug resistance	svRNAb down-regulates CYP3A4, a key enzyme in drug metabolism, causing drug resistance.	^69^,^171^

Abbreviations: AKT, protein kinase B; AML, acute myeloid leukemia; BCC, breast cancer cell line; CCC, cervical cancer cell; COX-2, cyclooxygenase 2; CSCC, cervical squamous cell carcinoma; CYP3A4, cytochrome P450 3A4; EBV: Epstein–Barr virus; ERKs, extracellular signal-regulated kinases; GAGE6, G antigen 6; GC, gastric cancer; HCC, hepatocellular carcinoma; Hela cell line, Henrietta Lacks cell line; JAK2/STAT3, Janus kinase 2 signal transducer and activator of transcription 3; MAPK, mitogen-activated protein kinase; MDS, myelodysplastic syndrome; ncRNA, non-coding RNA; NF-Κb, nuclear factor kappa light chain enhancer of activated B cells; NSUN2, NOP2/Sun RNA methyltransferase 2; PCa, prostate cancer; PD, Parkinson's disease; PI3K, phosphatidylinositol 3-kinase; PKR, protein kinase R; PSD-95, postsynaptic density protein 95; PSF, PTB-associated splicing factor/splicing factor proline–glutamine rich (PSF or SFPQ); PTB, preterm birth; RCC, renal cell carcinoma; SCs, stem cells; SQSTM1, Sequestosome 1; SRSF2, serine/arginine-rich splicing factor 2; SLE, systemic lupus erythematosus; TFEB, transcription factor EB; TGF β, transforming growth factor β; T2DM, type 2 diabetes mellitus; vtRNA, vault RNA.

Some data show approximately 97%–98% of the transcriptional output of the human genome is ncRNA.^7^, ^8^, ^9^, ^10^ Over the last few years, ncRNAs have attracted a lot of attention and can divide into common ncRNAs like rRNA, tRNA, small nuclear RNA, small nucleolar RNA, *etc*. New types of ncRNAs include microRNAs (miRNAs), long non-coding RNAs (lncRNAs), and circular RNA (circRNA).^11^, ^12^, ^13^ Furthermore, human cells express several cytoplasmic ncRNAs, including vault RNAs (vtRNAs) and brain cytoplasmic RNAs (BC RNAs,^14^ Y RNA,^15^ *etc*.). Interestingly, the physicochemical properties of RNA enable ncRNAs to perform a diversity of cellular functions through different mechanisms.^1^^,^^16^ They are involved in the regulation of many basic processes, including transcription, translation, RNA processing, mRNA turnover, DNA replication, genomics stability, gene expression, chromatin remodeling, and protein stability and localization.^1^^,^^16^, ^17^, ^18^, ^19^, ^20^ It should be noted that deregulated expression of ncRNA is a major cause of various pathological conditions.^21^

## Vault complex

### Vault components and structure

Interestingly, early findings revealed a highly conserved subset of ncRNAs derived from vault RNAs, so named because they were first discovered as part of the ribonucleoprotein complex known as the vault. Although they were first described 30 years ago in a Rome laboratory in 1986, vault RNAs are largely unknown and their molecular role is still under investigation.^21^^,^^22^ Vaults are revealed in partially purified fractions of human fibroblasts, mouse 3T3 cells, glial cells, and rabbit alveolar macrophages. These new ribonucleoprotein structures appear to be common in different cell types.^21^^,^^22^

The vaults are large cytoplasmic ribonucleoprotein particles with a sedimentation value of approximately 150 S.^23^^,^^24^ Based on the nature of the structure, the particle size of the vault was determined to be ∼55 × 30 nm, with a molecular mass of approximately 13 MDa (three times the size of a ribosome). The intact vault particle has a double symmetry, where each half-vault can begin in the shape of a flower with eight petals around a central ring. The remarkable conservation and wide distribution of the vaults suggest that their functions are essential for eukaryotic organisms and the structure of the particles must be important for their functions.^25^, ^26^, ^27^, ^28^

Moreover, the vaults are mainly located in the cytoplasm and can classify as cytoskeletal elements. In addition, part of the vault has been located on the cytoplasmic side of the nuclear membrane within or near the nuclear pore complex.^25^^,^^29^, ^30^, ^31^, ^32^, ^33^ However, their different morphology and subcellular distribution suggest an intracellular role^34^, ^35^, ^36^ and nucleocytoplasmic transport processes.^25^^,^^31^^,^^33^ The stoichiometric model of the vault particle suggests that it contains six copies of vtRNA, but these represent 5% of the total mass of the dome complex. Interestingly, 95% of vtRNAs are evenly distributed in the cytoplasm, while the remaining only 5% are directly associated with vault particles.^30^^,^^37^^,^^38^ It is important to point out that the vaults have a highly conserved morphology in eukaryotes and are widespread in eukaryotes.^27^ Vault RNA is an integral part of the large vault particle, a hollow barrel-shaped ribonucleoprotein (RNP) complex composed of multiple copies of three proteins: major vault protein (MVP), poly (ADP-ribose) vault polymerase (vPARP), and telomerase-associated protein 1 (TEP1). MVP is the main structural protein of the vault complex, accounting for about 70% of the mass of the particles, and self-assembles *in vivo* to form vault-shaped particles.^28^^,^^39^ It was recently shown that the absence of TEP1 completely disrupts the stable binding of vtRNA to purified vault particles and reduces the concentration and stability of the vault RNA. Thus, a novel role for TEP1 *in vivo* was discovered as an integral vault protein important for the stabilization and recruitment of the vault RNA into the vault particle.^10^^,^^40^ Furthermore, the precise role of this subunit in the telomerase complex is still unclear, except it is capable of binding telomerase RNA.^10^

### Genomic organization of multiple vault RNA genes and homologous evolution

**Figure 1:**
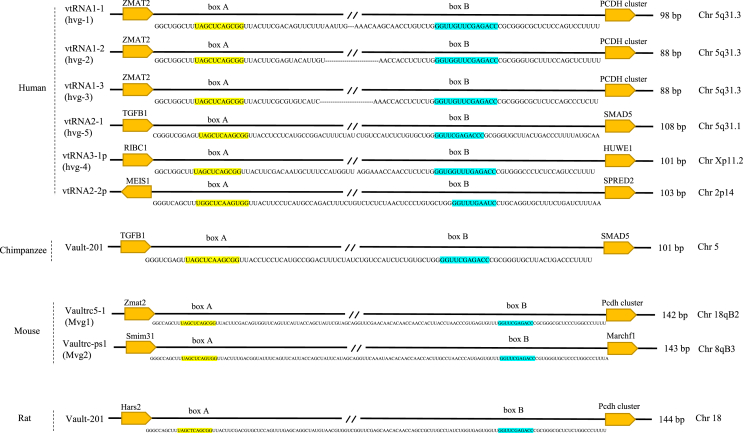
Overview of the evolution and location of vtRNAs genes. **(A)** In most mammals, the two syntenically vtRNA loci are highly conserved. Of the four human paralogs of vtRNA, hvg1-3 are located on the 5q31.3 chromosome between the *ZMAT2* and the *PCHD* gene. vtRNA2-1 locus is located between the *TGFB1* and *SMAD5* gene on chromosome 5q31.3. Furthermore, two vtRNA pseudogenes, vtRNA2-2p and vtRNA3-1p were annotated on chromosomes 2p14 and Xp11.2, respectively. Human vtRNAs are 88–150 nt in length, which are transcribed by RNA polymerase III and contain standard internal promoter elements of A and B. Also, most mammals, including mice and rats, have a single copy of the vtRNA gene on the *PCDH* locus. **(B)** The extended structure of vtRNAs of chimpanzees, mice, and rats. The positions of the internal transcription elements of boxes A and B are indicated, and the conserved sequences are highlighted.

Two vaults RNA loci are syntonically conserved in most mammals,^41^ and varying numbers of paralogs across animal kingdoms have been reported.^41^^,^^42^ Mechanistically, four human vtRNA paralogs have been identified and three related human vtRNA genes (hvg) have been described (hvg1-3; GenBank™ accession numbers: AF045143, AF045144, and AF045145).^10^^,^^30^ Human vtRNA gene sequences revealed that the vtRNA-1 locus located between the zinc-finger matrin-type 2 (ZMAT2) and the proto-cadherin cluster (PCHD) gene, which carries the genetic information for three hvg1-3 vault RNAs (vtRNA1-1, vtRNA1-2, vtRNA1-3) that are found in triplicate position 5q31.3 chromosome. The vtRNA-2 is a locus located between the genes encoding transforming growth factor beta 1 (TGFB1) and SMAD family member 5 (SMAD5) on chromosome 5q31.3, which encodes vtRNA2-1 (hvg-5).^30^^,^^37^^,^^43^ In addition, in the hg38 human genome assembly, two vtRNA pseudogenes, vtRNA2-2p and vtRNA3-1p (formerly hvg-4) were each annotated on chromosomes 2p14 and Xp11.2, respectively.^37^^,^^41^^,^^43^ Experimental evidence also suggests miR-886 is a vtRNA and not a canonical miRNA, as sequence alignment clearly identifies this sequence as either a vtRNA homolog or a vault complex associated molecule (later a homolog divergent of vtRNA2-1).^37^^,^^41^ Therefore, the revised name “vtRNA2-1” was approved by the HUGO Genetic Nomenclature Committee.^44^^,^^45^ Human vtRNA (hvtRNA) length ranges from 88 to 150 nt, with hvg-1 encoding 98-nt RNAs and hvg-2 and hvg-3 encoding similar 88-nt RNAs. All three genes are located in the 16 kb regions of chromosome 5, suggesting that these genes arose in tandem gene duplication.^30^^,^^32^ Nevertheless, hvg-4 seems to be unexpressed^32^ (Fig. 1).

The four genes transcribed by RNA polymerase III (Pol III) type 2 contain standard internal promoter elements of the A and B boxes with various external promoter elements and have a secondary core. Although the ring chain structure is well conserved,^37^^,^^41^ only the hvg1-3 gene contains external type-3 TATA, proximal, and distal sequence elements. In addition, it has been reported the sequences between A and B boxes are unique and affect the transcription efficiency. The sequence length among these elements is 41, 31, 31, and 44 bases in hvg-1, hvg-2, hvg-3, and hvg-4, respectively.^46^ Interestingly, vtRNAs isolated from different species have similar predicted secondary structures, despite differences in length, suggesting the association of vtRNAs with reservoirs is not random.^47^ The vault RNA sequence of a human, mouse, rat, and bullfrog was determined.^23^^,^^30^^,^^32^ Since vault RNA genes are only available for a few organisms, it is initially impossible to conclude whether there was a single gene or multiple copies.^23^^,^^46^

The human vtRNA-1 locus encodes three paralogues and their orthologs are also found in the phylogenetically close chimp (*P. troglodytes*). Instead, only two vtRNA-1 orthologs are encoded in the genome of other primates. This clue and sequence alignment analysis revealed that vtRNA1-2 and vtRNA1-3 are the result of recent gene duplication. Most mammals, including rats and related mouse models (*M. musculus*), have a single copy of vtRNA-1 at the *PCDH* locus.^41^ Significantly, rat vtRNA is 144 bases in length while bullfrog vtRNA exists as two highly related species of 89 and 94 bases. In addition, the rat vtRNA gene contains two B-box consensus elements separated by 34 bp. Rat and bullfrog vtRNAs contain sequences that correspond to the internal promoter elements of genes transcribed by RNA polymerase III.^23^^,^^24^

Particularly, in most mammals, the vtRNA-2 locus lacks or encodes only one paralog vtRNA, exception for the sloth genome (*C. hoffmanni*) which contains two paralogs, vtRNA-2 ^48^. Furthermore, the existence of vtRNA has been shown in the sea urchin (*Strongylocentrotus purpuratus*). However, the sequences were not determined.^49^ The vtRNA locus has been sequenced in other classes, including two copies of vtRNAs from *R. catesbeiana*,^23^ four paralogues in *H. sapiens*,^10^ and one each in *S. purpuratus*,^49^ T. brucei,^50^ *D. rerio* and *O. latipes*.^41^ In addition, the development of an iterative algorithm identified more than 100 potential vtRNA genes in the deuterostome genome.^41^

Interestingly, most mammals have a single vtRNA copy at the *PCDH* locus. In a few cases, notably in primates, guinea pig (*Cavia porcellus*), pika (*Ochotona princeps*), and shrew (*Sorex araneus*), they grow in a locally multicopy cluster.^41^ Besides, the dog (*Canis familiaris*) genome contains only a degraded pseudogene of vtRNA at the *PCDH* locus. Instead, there is a single alternative sequence (antisense direction) between *TGFB1* and *SMAD5*.^37^^,^^41^ Similarly, in gnathostomes, the functional vtRNA-1 gene is often located upstream of the *PCDH* cluster. However, in the Eutheria, there is a second locus with a functional vtRNA-2 gene between the *TGFB1* and *SMAD5* genes. In several eutherian lineages, only one of the two loci contains the vtRNA gene. This apparent complementation strongly supports the conclusion that the transcript of the vtRNA-2 locus is indeed an additional vtRNA.^37^ There are clear differences between vtRNA1-1 versus vtRNA1-2 and vtRNA1-3 sequences only within primates, suggesting that gene duplication occurred early in the primate lineage. Since the molecular function of vtRNA is still unknown, we cannot speculate on what might be the reason for this lineage-specific evolutionary pattern.^41^

### Vault RNAs functions

**Figure 2:**
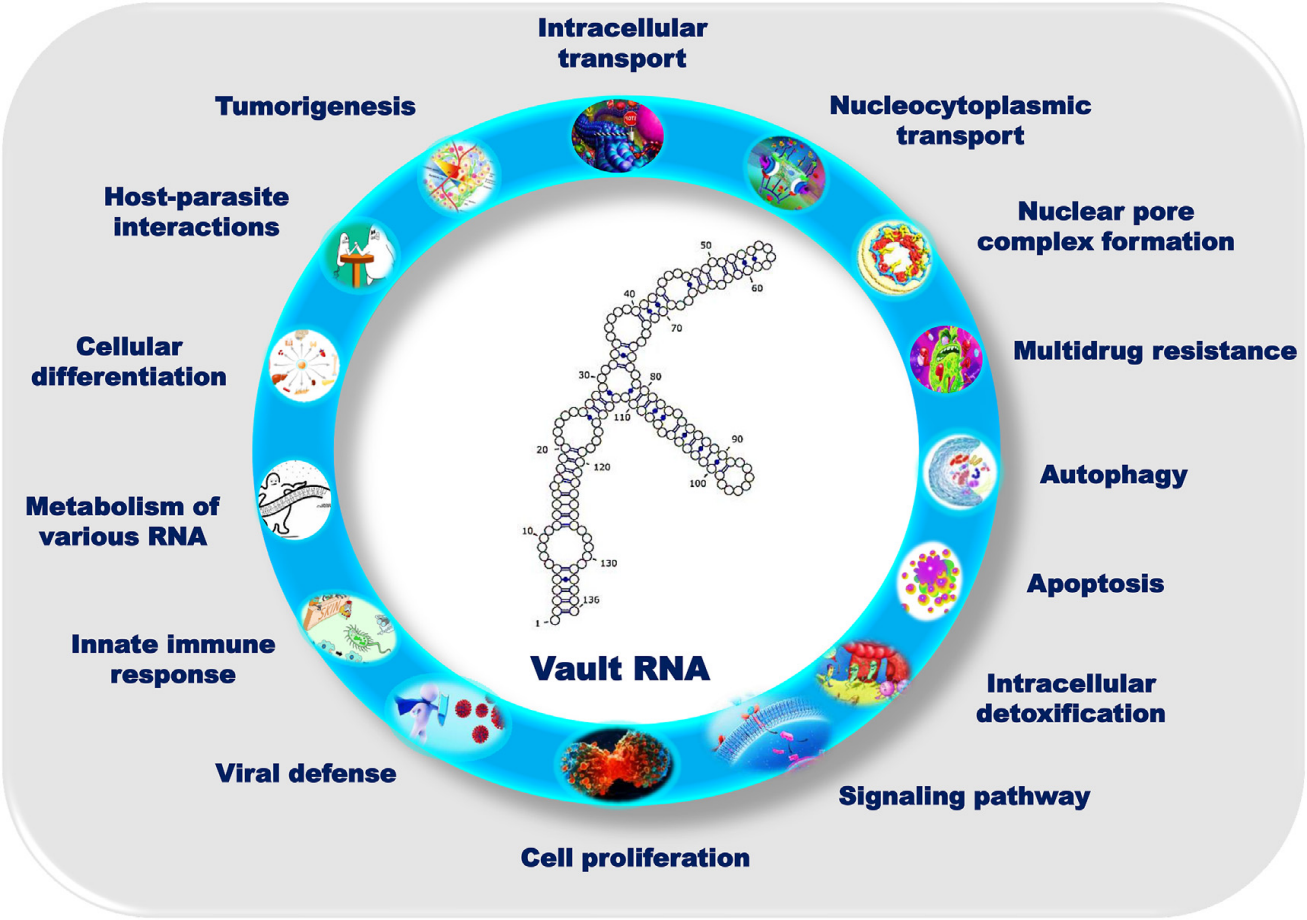
Possible mechanisms of vault RNAs are involved in different cellular processes and contribute to viral infections and tumorigenesis. Vault RNAs are a class of ncRNAs that contain stem-loop regions with well-conserved sequence patterns. They consist of only four members in humans and play fundamental roles in cells, possibly through RNA-ligand, RNA–RNA, or RNA-protein interactions. Accordingly, it has been proposed that vault RNAs are involved in cell proliferation and modulate the metabolism of multiple RNA classes: intracellular transfer, nucleocytoplasmic transport, regulation of central signaling pathways, regulation of cell communication, and more recently, nuclear pore complex formation. Furthermore, vtRNAs play an important role in the immune response, apoptosis, autophagy, detoxification processes, and multidrug resistance mechanisms. Most of our current knowledge of the functions of vtRNAs focuses on viral infections and cancer.

More recently, only a small fraction of vtRNAs is associated with the vault complexes,^30^^,^^51^ suggesting that the cellular function of ncRNAs is outside of the vault complex.^52^ The ends (5′ and 3′) of vtRNA form a stem-like structure, but the internal sequences of these RNAs are folded differently. These predictions indicate that vtRNA plays an important role in vault particle function, probably through RNA, RNA–RNA, or RNA-protein ligand interactions.^53^^,^^54^ Taken together, vtRNAs have been proposed to be involved in a variety of functions, including cell proliferation,^55^^,^^56^ nucleocytoplasmic transport, intracellular detoxification processes, and therefore a role in multidrug resistance (MDR),^30^^,^^53^^,^^57^, ^58^, ^59^, ^60^, ^61^, ^62^ signaling pathway,^63^^,^^64^ apoptosis,^65^^,^^66^ innate immune response,^67^ autophagy,^68^ which serve as miRNA precursors.^69^ Many of these results are consistent with the hypothesis that altered vtRNA expression is associated with tumorigenesis,^66^^,^^70^ viral defense,^52^ and modulation of the metabolism of different classes of RNA.^50^ Although the functional role of *F. hepatica* vtRNA is unknown, their selective packaging in the fluke extracellular vehicles (EVs) implies they may be involved in host–parasite interactions.^71^ Despite decades of dedicated research, the biological function of the vault particle or vtRNA has not yet been fully elucidated. It is evident that vtRNAs are vital regulatory molecules that orchestrate intracellular functions and can either drive or prevent pathophysiological processes (Table 1). The presence of this molecule in extracellular RNA circulating in liquid biopsies, serum,^72^^,^^73^ EVs from human samples, axons, and neurons,^74^^,^^75^ and shuttle RNA,^76^ makes it a promising candidate biomarker for the diagnosis and prediction of various diseases. This review summarizes the known functions of vtRNAs and their involvement in cellular mechanisms (Fig. 2).

### Vault RNAs and cancer

Cancer is an epigenetic and genetic disorder. However, the interaction between these two processes is unclear.^77^, ^78^, ^79^ Recent advances in genomic technology have identified thousands of ncRNAs in the human genome. Although the role of ncRNAs in cancer epigenetics has been demonstrated, most have not been assigned a role.^80^^,^^81^ ncRNAs have emerged as key players in cancer initiation and progression,^82^, ^83^, ^84^ therefore, their clinical value is the subject of intense investigation.^44^^,^^85^ An interesting observation regarding the role of vtRNA is the association of vtRNA with the vault complex in several human cancer cell lines.^32^^,^^53^^,^^66^^,^^70^

## DNA methylation status of vault RNAs

### Hematopoietic diseases

Acute myeloid leukemia (AML) and myelodysplastic syndrome (MDS) belong to a heterogeneous group of clonal hematopoietic diseases. MDS and AML are often associated with deletions at chromosome 5q and aberrant DNA methylation patterns, including hypermethylation, indicating the presence of important tumor suppressors at this locus.^86^^,^^87^ In normal hematopoiesis, vtRNA shows differential methylation between different hematopoietic cell populations, suggesting that allele-specific methylation may occur during hematopoiesis.^87^ Treppendahl and colleagues demonstrated that AML patients with deficient vtRNA2-1 methylation had significantly bettered outcomes than patients with mono or biallelic methylation. In other words, this probably implies that a higher gene dose influences the outcomes. Consistent with the result of this study, the methylation is inversely proportional to vtRNA2-1 expression, and 5-azanucleosides induce vtRNA2-1 and reduce phosphorylated RNA-dependent protein kinase (pPKR) whose activity is regulated by vtRNA2-1. Since pPKR promotes cell survival in AML, the data are consistent with vtRNA2-1 as a tumor suppressor in AML.^86^ In addition, vtRNA1-3 promoter hypermethylation is frequent in lower-risk MDS patients and is associated with a decreased overall survival.^87^

### Gastric cancer

Lee et al investigated that nc886 suppresses cell proliferation when ectopically expressed in gastric cancer cells by suppressing oncogenes such as FOS, NF-κB, and MYC, so nc886 CpG hypermethylation and corresponding down-regulation are significantly associated with lower survival rates in gastric cancer patients.^55^ The nc886 RNA is a confirmed tumor suppressor ncRNA that acts by down-regulating the cancer survival factor pPKR.^86^^,^^88^ The pre-miR-886 was further found to be physically associated with PKR, an interferon-inducible and double-stranded RNA-dependent kinase.^89^ Deletion of pre-miR-886 reduces cell proliferation through activation of PKR and its downstream pathways, phosphorylation of eIF2α, and the NF-κB pathway. Pre-miR-886 was deleted in some cancer cell lines and clinical samples.^89^ However, valid data based on the precise identification of nc886 for its functional cellular function as a tumor suppressor has not yet been provided.

### Hepatocellular carcinoma

Similarly, hepatocellular carcinoma (HCC) patients had worse outcomes when the vtRNA2-1 promoter was differentially hypermethylated in tumor tissues compared with adjacent normal tissues. In addition, vtRNA2-1 promoter methylation is increased in stage III HCC compared with stages I and II. These results indicate that differential hypermethylation of the vtRNA2-1 promoter is significantly associated with tumor recurrence.^90^

Based on the above observations regarding the involvement of vtRNA in the pathology of various tumors, it can be concluded that vtRNAs are proposed as a new type of ncRNA that acts as a tumor suppressor (TSG) gene which inhibits PKR and *vice versa*. In contrast, some reports have shown that vtRNAs act as oncogenes in tumor progression, development, and invasion. Therefore, it is a potential prognostic biomarker and therapeutic target for tumors.

## Vault RNAs and non-communicable diseases

### Age-related neurodegenerative disease

Parkinson's disease (PD) is known to be the second most common age-related neurodegenerative disease. Nowadays, it is most accurately diagnosed in the advanced stage with a series of motor deficits preceded by a litany of non-motor symptoms that appear over years or decades.^91^^,^^92^ Notably, recent studies found that blood-based gene expression or epigenetic biomarkers exist across a range of PD diseases and are highly desirable and non-invasively detectable markers.^91^^,^^93^

Recently, Henderson et al reported hypermethylation of 15 CpG sites encompassing the vtRNA2-1 gene in the whole blood of Parkinson's disease.^91^ A previous study by Miñones-Moyano et al also showed that a small non-coding RNA considered as a fragment of vtRNA2-1 (svtRNA2-1a) was up-regulated in the amygdala and frontal cortex of patients with advanced PD. Moreover, a double up-regulation was also found in preclinical cases of Alzheimer's disease. The expression patterns of svtRNA2-1a during human brain development and aging and the deleterious results of mimicking svtRNA2-1a overexpression in neurons further suggest that low levels of svtRNA2-1a may be important for neuronal maintenance. These results proposed that early dysregulation of svtRNA2-1a in PD may lead to disruption of gene expression networks, metabolic disturbances, and cellular dysfunction.^94^

### Synapse formation

Recent evidence has been provided for a novel role for vtRNA in synapse formation. New studies show that mouse vtRNA (mvtRNA) promotes synapse formation by regulating mitogen-activated protein kinase (MAPK) signaling. mvtRNA binds to and activates mitogen-activated protein kinase 1 (MEK1) in dendrites, enhancing MEK1-mediated extracellular signal-regulated kinase activation, thereby regulating synapse generation.^95^ In addition, the expression of hvtRNA1-1, but not hvtRNA2-1, increases the expression of synaptic marker proteins, extracellular signal-regulated kinase (ERK) phosphorylation, and the number of double-positive postsynaptic density protein 95 (PSD-95), and synapsin I to a similar extent as mvtRNA. According to this knowledge, hvtRNA1-1 modifies the modulation of the MAPK signaling pathway to improve synapse formation.^96^ Therefore, a detailed understanding of the mechanisms of synapse formation will allow further elucidation of the pathogenesis of neurodevelopmental disorders. Furthermore, a better understanding of the signaling complexes involved in vault complexes could lead to the development of new therapeutic strategies for neurological disorders.^95^

### Type 2 diabetes

Glucagon-like peptide-1 (GLP-1) is an incretin that plays a critical physiological role in glucose homeostasis, which can stimulate the secretion of insulin produced by the intestine during meals and keeps the pancreatic β-cells healthy and proliferating.^97^ The therapeutic efficacy of GLP-1 analogs in type 2 diabetes (T2DM) is 50%–70%. In support of this, the discovery of genetic biomarkers is still needed to predict the therapeutic efficacy of GLP-1 analogs for treatment. Today, the glycemic response to GLP-1 analog therapy is associated with the methylation status of the vtRNA2-1 promoter and rs2346018 polymorphism. Furthermore, hypomethylation of vtRNA2-1 (<40% methylation) is significantly associated with a poor glycemic response to GLP-1 treatment.^98^

### Glomerulonephritis

IgAN (IgA nephropathy) is the most common form of primary glomerulonephritis worldwide and has a strong genetic component.^99^^,^^100^ In this case, DNA methylation may also be an important factor influencing the disease.^100^^,^^101^ Sallustio et al. (2016) identified for the first-time specific regions of DNA that were abnormally methylated in IgAN patients, leading to reduced TCR signaling strength of CD4^+^ T cells and their abnormal response and activation, resulting in cellular imbalance. In addition, hypermethylation of miR-886 precursors reduced CD4^+^ T cell proliferation and led to overexpression of transforming growth factor (TGFβ) upon TCR stimulation. This study reveals a novel molecular mechanism underlying the aberrant CD4^+^ T cell response in IgAN patients.^100^

### Sensitivity to ultraviolet B

Unprotected advertising to ultraviolet (UV) A and B can damage the DNA of pores and skin cells, causing genetic defects or mutations that can result in pores, skin cancer, and various other pores and environmental skin conditions (including premature aging). UV rays can also harm your eyes, including cataracts and eyelid pores, skin cancer, and a variety of environmental pores and skin conditions.^102^, ^103^, ^104^ Under changes in pores and skin cells induced by UVB radiation, down-regulation of nc886 results in depleted phosphorylation of PKR by MAPKs and increased expression of proinflammatory cytokines, matrix metalloproteinase-9 (MMP-9), type IV collagenase, and cyclooxygenase (COX-2), which in the end boost up inflammatory responses and pores and skin aging.^105^

To sum up, these observations highlight the important role of vtRNAs in the progression of non-infectious diseases through various mechanisms such as PKR-specific binding and signaling pathways, and this mechanism opens new clinical perspectives for the treatment of non-infectious diseases.

### Vault RNAs and viral infection

In human viral diseases, infection of host cells is a tightly controlled process, with a temporally regulated expression of viral and cellular genes.^106^^,^^107^ Recently, viral infection has been shown to alter RNA expression profiles at the ncRNA level.^37^^,^^108^ For example, the most up-regulated ncRNAs in the presence of Epstein–Barr virus (EBV) were three host-encoded vtRNAs.^22^ vtRNA promoted viral replication by inhibiting PKR activation and subsequent responses to antiviral interferon. In addition, increased vtRNA expression was required for efficient inhibition of PKR by NS1 during IAV infection. Viral activation of host vtRNA serves as a mechanism supporting inhibition of PKR signaling, representing a viral strategy to evade host innate immunity.^52^^,^^109^ It is therefore tempting to speculate that the vault complex may be an evolutionary remnant of a primitive viral symbiont of eukaryotic cells.^37^ Evidence from previous studies suggests the viral infection induces vault RNA expression^43^^,^^110^ in *in-vitro* models for several viral families, including γ-herpesviridae (herpes simplex virus 1), paramyxovirus (EBV), and influenza-A virus.^37^^,^^43^^,^^52^ Therefore, the higher expression of vault RNAs can cause a higher viral load.^111^

### How does vtRNA regulate protein kinase R function?

Protein kinase R (PKR) is an interferon (IFN)- and double-stranded RNA (dsRNA)-activated Ser/Thr protein kinase that is an important part of host innate immunity to viral infection.^52^^,^^112^ Upon activation, PKR phosphorylates its own and downstream substrates, including eukaryotic initiation factor subunit 2α (eIF-2α) and IκB.^113^^,^^114^ Phosphorylated EIF2α dramatically inhibits viral protein synthesis, blocking viral replication.^51^^,^^113^ Furthermore, PKR promotes the activation of nuclear factor kappa B (NF-κB) through phosphorylation of IκB.^114^ Transcription factor NF-κB positively regulates IFN gene transcription and promotes the expression of IFN-stimulated genes (ISGs).^115^^,^^116^ Influenza A virus (IAV) infection does not activate PKR.^117^ In contrast, IAV inhibits PKR activity through the virally encoded nonstructural protein (NS1) and the cellular protein p58IPK.^118^, ^119^, ^120^ Previous studies have shown that AVA sequesters and activates p58IPK from heat shock inhibitory protein 40 (Hsp 40), and activated p58IPK inhibits PKR dimerization and phosphorylation through direct interactions between these molecules.^121^^,^^122^ Furthermore, PKR activity can be significantly induced by NS1-deleted virus.^123^ The viral IAV NS1 protein has been shown to be an inducer that initiates vtRNA up-regulation. The vtRNA silencing animal model showed significantly more resistance to IAV infection, as evidenced by amelioration of acute lung injury and splenic atrophy, thereby increasing survival. Interestingly, vtRNA promoted viral replication by inhibiting PKR activation and subsequent responses to antiviral interferon. In addition, increased vtRNA expression was required for efficient inhibition of PKR by NS1 during IAV infection.^123^

However, the inhibiting mechanism of PKR activation by NS1 is still unknown. NS1 can cleave PKR dsRNA,^118^^,^^123^ but the affinity between NS1 and dsRNA is low.^124^ Furthermore, the requirements for the NS1 RNA-binding domain and the direct interaction between NS1 and PKR are controversial based on reports from several research groups.^120^^,^^125^, ^126^, ^127^ Of note, in a previous study, nc886 effectively competes with dsRNA for binding to PKR, attenuating dsRNA-mediated PKR activation, thereby suppressing the interferon response. These data suggest that nc886 sets a threshold for PKR activation and triggers activation only when the virus is infected. As discussed above, PKR is activated by various RNA species as well as by actual dsRNA. In the absence of nc886, PKR can be activated by measuring basal levels of this RNA, which can have lethal effects on host cells.^128^

### Vault RNAs and autophagy

Macroautophagy (referred to further as autophagy) is an evolutionarily conserved degradative pathway activated by cellular stress and responsible for the recognition, recycling, elimination, and degradation of intracellular components, organelles, and pathogens in membrane vesicles called autophagosomes.^129^^,^^130^ In this excretory cycle, after engulfing the cargos, the autophagosomes approach and fuse with the lysosomes, breaking down their contents to supply amino acids, lipids, and nucleotides for the anabolic desires of the cells.^68^ Autophagy removes intracellular substrates ranging from simple ubiquitinated proteins to large proteotoxic aggregates. Human selective autophagy receptor p62 (also known as sequestosome protein 1 (SQSTM1), zeta-interacting protein (ZIP), or orphan receptor co-activator (ORCA)) recruits them into double-membrane vesicles and regulates intracellular load to maintain homeostasis.^131^, ^132^, ^133^, ^134^ Intracellular pathogenic bacteria and viruses have evolved different defense strategies against autophagy, possibly related to vtRNAs levels.^68^

### Mechanism of vtRNA to modulate autophagy signaling

Recent studies reported the depletion of vtRNA1-1 led to the accumulation of mature autophagosomes, indicating increased autophagic flux as one of the major vtRNAs interacting with p62.^68^,^109^ Moreover, up-regulation of vtRNAs levels appears to be a highly effective way to avoid autophagy-targeted viral degradation and subsequent MHC class II antigen presentation.^135^ Büsche et al. found that small non-coding vtRNA1-1 bind directly to p62 and regulate p62 oligomerization, thus functioning in autophagy.^68^^,^^136^ Similarly, a recent study in Huh-7 cells and mouse cells reported that vtRNA1-1 can help regulate autophagic flow through direct interaction with the autophagy receptor protein SQSTM1/p62 ^68^. Additionally, it was later found the vtRNA1-1 depletion results in the down-regulation of transcription of several catabolism-related genes, including SQSTM1/p62. These data indicate that the reduced expression of SQSTM1/p62 is transcriptionally controlled and does not necessarily depend on the interaction with vtRNA1-1.^68^^,^^137^, ^138^, ^139^ This reboregulation affects the aggregate state of p62 and therefore its autophagic degradation and its function as a selective receptor for autophagy.^138^^,^^139^

In addition to its role as an autophagy receptor, p62 provides a platform for important cell signaling pathways.^140^, ^141^, ^142^, ^143^ It will be interesting to explore worldwide whether the depletion of vtRNA1-1 or the expression of p62 mutants without RNA binding influences other cell signaling pathways.^144^ The p62/vtRNA1-1 interaction provides a paradigm for the interference of a rib-regulator RNA with protein–protein interactions.^144^ It has also been shown in detail that the Phox and Bem 1 domain (PB1) and the adjacent linker region (p62_1–122_) are necessary and sufficient for maximal and specific binding of vtRNA1-1. Mutation analysis indicates that K7 and R21 are essential amino acids required for binding to RNA.^144^ In addition to the previously known role in the oligomerization of p62.^145^ This result identifies these two residues as cornerstones of riboregulation by which vtRNA1-1 inhibits p62 oligomerization and hence autophagy.^144^ Furthermore, Ferro et al. showed that vtRNA1-1 knockdown caused alkalinization of intraluminal lysosomal pH, leading to a marked change in lysosome-mediated clearance proteolytic and degradative activity.^137^

### vtRNAs and apoptosis

Apoptosis is a sequential sequence of cell death that occur regularly to ensure a homeostatic balance between the rate of cell formation and cell death. However, dysfunction of this compensatory function can contribute to abnormal cell growth/proliferation or autoimmune diseases, *etc*.^146^^,^^147^ An increasing number of studies have shown that ncRNAs play an important role in various cellular processes, including proliferation and apoptosis.^148^^,^^149^ For the first time, Amort and colleagues have shown general apoptotic resistance after overexpression of vtRNA1-1 in human B cells infected with Epstein-Barr virus (EBV).^66^ Here, researchers individually identified latent membrane protein 1 (LMP1) that stimulates vtRNA1-1 expression in BL2 cells as a trigger for NF-κB-dependent up-regulation of vtRNA1-1. Ectopic expression of vtRNA1-1, but not other vtRNA paralogs, results in increased viral establishment and reduced apoptosis, leaving cells vulnerable to efficient EBV infection.^66^

Another aspect was also revealed, that the knockdown of the main vault protein does not affect these phenotypes, demonstrating that vtRNA1-1, not the vault complex, contributes to a general resistance to cell death, a function located in the central domain of vtRNA1-1. This study describes an NF-KB-mediated role of vtRNA1-1 in inhibiting both extrinsic and intrinsic apoptotic pathways.^66^ In addition, another group of researchers showed in HeLa knockout cell lines that prolonged starvation caused increased levels of apoptosis in the absence of vtRNA1-1, but not in vtRNA1-3 knockout cells.^70^ Next-generation deep sequencing of mRNome identified the phosphatidylinositol 3-kinase/protein kinase B (PI3K/Akt) pathway and the ERK1/2-MAPK cascade, of which two critical signaling axes are dysregulated under starvation conditions of mediated cell death in the absence of vtRNA1-1.^70^ It should be noted that the expression of the vtRNA1-1 mutant identified a short 24-nucleotide stretch of the central domain of vtRNA1-1 as essential for successfully maintaining resistance to apoptosis.^70^ Similarly, an earlier study from Kong et al. indicates that vtRNA2-1-5p has oncogenic activity linked to cervical cancer progression and the vtRNA2-1-5p also directly targets p53 expression.^150^ Surprisingly, inhibition of vtRNA2-1-5p has been shown to increase Bcl-2-associated protein *X* (Bax) expression and apoptotic necrobiosis in cervical cancer cells.^150^ Inhibition of vtRNA2-1-5p reduced invasion, proliferation, and tumorigenicity of cervical cancer cells while increasing apoptosis and p53 expression.^150^ These results imply that vtRNA2-1-5p may be a direct regulator of p53. Therefore, it plays a crucial role in the apoptosis and proliferation of cervical neoplastic cells and will be a potential target in the treatment of cervical cancer.^150^

Similarly, Lei et al. showed that overexpression of nc886 promotes proliferation and invasion of A-498 cells and inhibits cell apoptosis, while deletion of nc886 produces the opposite effects. Additionally, nc886 could activate the Janus kinase 2 signal transducers and the transcription activator 3 (JAK2/STAT3) in A-498 cells. AG490 (tyrphostin), a JAK2 inhibitor, may attenuate the effect of nc886 on cell proliferation, apoptosis, and invasion. This study could represent a useful therapeutic target for renal cell carcinoma (RCC).^151^

### Vault RNAs and cellular differentiation

Stem cells (SC) are unique cells that have an inherent ability to self-renew or differentiate.^152^^,^^153^ These two fateful decisions are tightly regulated at the molecular level by complex signaling pathways. It has been hypothesized the regulation of signaling networks that promote self-renewal or differentiation is largely influenced by the action of transcription factors.^152^^,^^154^ However, small ncRNAs, such as vtRNAs, and their post-transcriptional modifications (epithrescriptome) have emerged as additional regulatory layers that play an essential role in SC fate decisions. Since epitranscriptoma and associated proteome are essential in SCs, they would be considered new tools for the spread of SCs for regenerative medicine.^152^

The skin is a versatile tissue supported by numerous SCs some of which are unipotent such as epidermal SCs while others like bulge SCs are multipotent.^155^^,^^156^ In NOP2/Sun RNA Methyltransferase 2 (NSUN2) knockout mice, SCs bulging into the hair follicle show delayed differentiation when stimulated during the start of the hair cycle.^155^ Normally, NSUN2 expression peaks in bulging SCs when they enter anagen, the growth phase of the hair cycle. When NSUN2 ablates in the skin, resting and protruding SCs still accumulate at anagen onset, leading to a general delay in differentiation.^155^ Similarly, isolated bulge SCs and epidermal SCs from NSUN2 knockout mice show difficulty in *in-vitro* differentiation.^155^ Accumulation of 5′tRF disrupts migratory responses in epidermal cells, which may arguably be the underlying mechanism limiting differentiation.^155^^,^^157^ In humans, NSUN2 targets vtRNA1-1 with a single m5C at C69.^158^^,^^159^ Human epidermal progenitors *in vitro* inhibit differentiation by promoting vtRNA1-1 methylation-dependent processing in small vault RNAs (svRNAs) bound to the RNA-induced silencing complex (RISC).^160^^,^^161^ Methylated vtRNA1-1 prevents the binding of serine/arginine-rich splicing factor 2 (SRSF2) to its purported binding site covering methylated C69. Without the protection conferred by SRSF2 binding, vtRNA1-1 is preferably cleaved into svRNA4, which in turn inhibits epidermal differentiation.^160^ Exactly how svRNA4 blocks skin differentiation is unclear; however, predicted targets of svRNA4, such as Ovo-like transcriptional repressor 1 (OVOL1), could be its mode of action.^161^ Indeed, this may be the case, as low levels of OVOL1 are needed to stop epidermal differentiation.^160^^,^^161^

Currently, m5C is the only post-transcriptional RNA modification associated with skin SC functions. Further studies are needed to determine if a different epitranscriptome signature plays a role in skin SCs.^152^ The presence and absence of RNA modifications regulate RNA metabolism by modulating the binding of writing, reading, and erasing proteins.^160^^,^^162^^,^^163^ However, how 5-methylcytosine (m5C) recruits or repels RNA-binding proteins is largely unknown. Methylation of cytosine 69, in vtRNA1-1, occurs frequently in human cells, is mediated exclusively by NSUN2, and determines the processing of vtRNA1-1 into svRNA. Serine/arginine-rich splicing factor 2 (SRSF2) has been identified as a novel vtRNA1-1 binding protein that antagonizes vtRNA1-1 processing by binding the unmethylated form with higher affinity. Both NSUN2 and SRSF2 orchestrate the production of different svRNAs.^160^ Finally, the research group revealed a direct role for m5C in the processing of vtRNA1-1, including SRSF2, and plays a critical role in the regulation of the epidermal differentiation process.^160^ Interestingly, identifying the mechanism of molecules involved in the cell differentiation process may be effective for better clinical application of cell differentiation-based therapies in human diseases.

### Vault RNAs and drug resistance

Multidrug resistance (MDR) is the most common cause of chemotherapy failure to treat cancer. Multiple mechanisms are responsible for mediating MDR, including overexpression of transmembrane transporters such as ATP binding cassette (ABC) transmembrane transporters such as P-glycoprotein (Pgp) and multi-drug resistance-associated protein (MRP1), which act as drug efflux pumps.^30^^,^^164^, ^165^, ^166^, ^167^, ^168^ Furthermore, MVP is effective as an independent prognostic factor in predicting tumor response to standard chemotherapy and survival in patients with advanced stages of many cancers.^169^ However, no vault components have been identified to directly be involved or interact with the chemotherapeutic compounds.^53^

According to the surprising data obtained in a recent study, safe levels may be a good predictor of drug resistance, as their up-regulation alone is not sufficient to confer the drug-resistant phenotype. This indicates a need for extra factors to vault-mediated MDR. Vault up-regulation may be necessary but not sufficient for multidrug resistance.^48^ Previous studies showed the components that bind safely to cancer cells are resistant to chemotherapy drugs.^30^^,^^48^^,^^170^, ^171^, ^172^ Importantly, another previously discovered aspect of Persson et al. study found that svRNAb down-regulates cytochrome P450 3A4 (CYP3A4), a key enzyme in drug metabolism. These findings expand the repertoire of small regulatory RNAs and, for the first time, assign functions to vtRNAs that may help explain the relationship between vault particles and drug resistance.^69^ CYP3A4 is the most abundant cytochrome P450 enzyme in the human liver and intestine and contributes to the metabolism of >60% of all drugs.^171^^,^^173^ The hepatic expression levels of CYP3A4 vary widely between individuals.^171^ However, the detailed regulatory mechanisms of CYP3A4 expression remain unclear. Additionally, a negative correlation was found between svRNAb and CYP3A4 in human tissue samples.^171^

Furthermore, luciferase assays in human liver cancer G2 (HepG2) cells confirmed that svRNAb regulates CYP3A4 expression by directly targeting CYP3A4 and interacting with a validated binding site in the CYP3A4 3′UTR.^171^ The results provided insight into changes in CYP3A4 expression between persons and provided a new way to tailor individualized drug therapy. Moreover, this study provided a novel regulatory mechanism of svRNAb in MDR cells.^171^ New data suggest that vtRNA may be involved in sequestering chemotherapeutic compounds, preventing the drugs from reaching their target sites.^56^^,^^62^ Higher levels of vtRNA1 expression appear to be involved in the drug resistance phenotype than mitoxantrone.^56^^,^^62^ Interestingly, inhibition of vtRNA expression by RNA interference in resistant human cancer cells to chemotherapy resulted in a progressive decline in resistance.^62^

Consistent with the above results, vtRNA was found to be significantly superior to MVP by cloning the human gene for vtRNA and carefully determining the levels of MVP and vtRNA in MDR cells. Measurements of vault particle deposition in multiresistant cells showed a 15-fold increase in vault synthesis. The vaults synthesis directly related to multidrug resistance supports a direct role of vaults in drug resistancen.^30^ Besides, another group of researchers showed inhibition of nc886 increased chemosensitivity, induced apoptosis, and suppressed MVP protein expression, a key regulator of drug resistance in cervical cancer cells. Furthermore, they showed E2F transcription factor 1 (E2F1) promoted nc886 transcription and can promote drug resistance in cervical cancer cells by altering MVP expression.^89^^,^^174^

Ferro et al. described a new insight into the survival-promoting role of vtRNA1-1 *in vitro* and *in vivo*. Knockdown (KO) of vtRNA1-1 in human HCC cells inhibits transcription factor EB (TFEB) nuclear translocation leading to dysfunction of the lysosomal compartment, resulting in the down-regulation of coordinated lysosomal expression and regulation (CLEAR) network genes. In addition, the depletion of vtRNA1-1 increased the activation of the MAPK cascades responsible for inactivating TFEB, MAPK1/ERK2, and MAPK3/ERK1. Finally, the study group showed the absence of vtRNA1-1 reduced drug hemolysis and significantly inhibited tumor cell proliferation *in vitro* and in mouse models. These results demonstrate the role of vtRNA1-1 in maintaining the function of the intracellular catabolic compartment in human hepatocellular carcinoma cells and highlight its importance in lysosome-mediated chemotherapy resistance.^137^

## Conclusion and perspectives

Most human RNA transcripts are not known to encode proteins, and ncRNAs are known to regulate cellular physiology and cellular functions. Rapid advances in network biology indicate a highly conserved evolutionary subset of ncRNAs are represented by vtRNAs, so named because they were first discovered as part of a larger ribonucleoprotein complex known as the vault. Although vtRNAs were first described 30 years ago, their molecular functions are still under investigation. In humans, the vtRNA-ncRNA family, consisting of only four members, plays a vital role in cells. As described in this review, vtRNAs are involved in cell proliferation, metabolic regulation of different classes of RNA, intracellular transport, nucleocytoplasmic transport, nuclear pore complex formation, regulation of central signaling pathways, and cellular communication. In addition, vtRNAs play a crucial role in immune responses, influence apoptosis, and autophagy, and are involved in detoxification processes, and mechanisms of resistance to many drugs. Most of our current knowledge of the functions of vtRNAs focuses on viral infections and cancer. What are the general biological principles of regulation of cellular processes by vtRNA? The relationship between vtRNA and cellular machinery requires further investigation, as the role of vtRNA is associated with vault particles, and the function of vault particles is not well understood. Interestingly, infection by several virus families, including γ-herpesviridae, paramyxoviruses, and influenza A, elicits a high expression of vtRNA. On the other hand, high expression of vault RNAs has been shown to lead to increased viral loads. Viruses are known to hijack cells and cellular replication machinery to maximize viral replication while inhibiting cellular defense mechanisms. We wonder if the vtRNA regulatory axis could be an important aspect of the molecular pathogenesis mechanism of the new lethal viral pandemic SARS-CoV-2. A recent study reported that SARS-CoV-2 infection induces autophagy and apoptosis in human microvascular bronchial endothelial and epithelial cells^175^ and can bypass antiviral pathways induced by viral double-stranded RNA, including PKR and interferon (IFN) signaling.^176^, ^177^, ^178^ So, could these strong observations point to a key role for vtRNAs in SARS-CoV-2 infection? Furthermore, the details on the identity, role, and mechanism of action of vtRNA will advance the mechanistic understanding of the molecular mechanisms of the RNA-directed in pathological conditions, as well as the development of new RNA-based diagnostic and therapeutic strategies in viral infections and unknown diseases. However, these results are still limited and future research in this area may lead to a better understanding of previously discovered processes.

## Conflict of interests

The author declares that there is no conflict of interests.
